# 

**Published:** 1887-03

**Authors:** 


					﻿VOL VII.
MARCH, 1887.
NO. 3.
JPHE
pDMtEOPATHlC pHY^lClAJI,
A MONTHLY JOURNAL OF MEDICAL SCIENCE.
“ He is a freeman whom truth makes free., and all are slaves, beside.”
u Seek the Truth : come whence dt may, cost what it will.”
EDITED BY
Edmund j. lee, m. d.,
AND
WALTER ML. JAMES, ML. ID.
PHILADELPHIA :
No. 1133 SPRUCE STREET.
NHW YOiRiti
A. L. CHATTERTON & COMPANY, Publisher’s Agents.
ALFRED HEATH & CO., Sole Agents fob Great Britain,
114 Ebury Street, Eaton Square.
yearly Subscription, $2.50, invariably in advance. Single numbers, 25 cts.
- Entered at the Philadelphia Post-Office as Second* Class Mail Matter.
				

